# Answers to Correspondents

**Published:** 1903-08

**Authors:** 


### CORRESPONDENCE.

### Answers to Correspondents.

RECENTLY there have come addressed to the editor of this JOURNAL, several communications of varying interest, which are here given, care being taken to suppress the names signed, as is done by all well-regulated publications. The answers are written by the reviewer, who gets what he terms "the morgue journals," and who has been driven thereby to a flippancy far beyond his years. The first gem in this unique setting of epistolary literature follows:

BUSINESS SUCCESS.

*Esteemed Coworkers:*

Our business having grown to such proportions as to demand more commodious quarters, we have bought the three-story building at —— ——— Boulevard, and would ask you to change our address on your books to that number. Hoping that your publication is equally prosperous, we are,

Very sincerely yours,

——— ————

Your attention is requested to the markt part of enclosed reprint.

*Answer.*—We are pleased to learn that your business has grown to such proportions, but we must beg to remind our esteemed co-worker, that our own business is able to sit up and take nourishment, and that we are contemplating the erection of a six-story building, with jade trimmings and a circular storm door for winter use, which we expect to erect on a private boulevard, admission to be by invitation and subscription, which we intend shall be named Rue d'Medicine. Trusting that your publication will become equally prosperous, we are, etc., etc.

P. S.—Your typewriter lady is very careless; we note that she spells marked "markt." If Dr. Gould should see that—but why anticipate! He has troubles of his own just now.

The following from an esteemed fellow editor, requires more space, which is cheerfully given for the sake of the suffering:

MORE BUSINESS SUCCESS.

*Fellow Editor:*

*Greeting*—Our fiscal year ends June 30th of each year. At the closing of the books for the year just completed, I was delighted to find (for the first time in our history, except it may have been in the early years before much advertising patronage came to us), that *our subscription receipts for the year exceeded our advertising receipts!* This, we understand, is phenomenal, particularly for a $1 monthly. This was not due to decline in our advertising receipts, but to the increase in our subscription returns.

I have become convinced that both for our financial interests, and for the dignity and independence of journalism, it is our duty to collect our subscription accounts as closely as possible. Doctors are willing to pay for what they really want. I am trying to get doctors to *pay promptly* for the periodicals they really want, and decline to receive all others. We do not wish to send our journals to doctors who don't intend to pay. We have been too careless about this for our own good. I prepared a paper on this subject, for the meeting in ——————. I inclose a copy herein. I prepared it in the interest of straight-forward, legitimate medical journalism. I trust that it has served, and will serve its purpose. Are you not tired of giving away literature? I am. Is it not worth its price? Then let us get it. If we all collect our subscription accounts, it will be better for us all financially, better for journalism, and better for the profession, for literature that is paid for is more highly prized, and the editor who is *paid* can produce better literature.

Sincerely, your co-worker,

—————— ——————.

*Answer.*—This showing is really phenomenal and we extend our congratulations. Of course, we do not doubt the statements shown by the books of our fortunate fellow editor. Educated books are a luxury, which few of us can afford in these days of competition in medical journals; yet we have long had our eye on a set of self-winding books, called the Autoequilibrist, because they balance themselves. All one has to do is to set a dial and get almost any result one wants. We have been experimenting with a sample set and have had some really remarkable results. Once, when the fiscal year was supposed to end on Christmas eve, the combination was set and after severe work on the part of the books, we found that our advertising receipts had trebled in cash, while the actual advertising space had shrunk to one-half its original quantity. This was all the more remarkable, because the checks for the advertising at the increased prices necessary to balance the books were found pinned to each account, all properly endorsed and ready for banking. On communicating with the advertisers, the startling fact was established, that the amounts had already been drawn from their bank accounts and receipted bills received. It is a great labor-saving device.

As to the collection of subscription accounts we must beg to be excused from taking part in so delicate a subject as the matter apparently is with our fair correspondent. The JOURNAL has a subscription list and a fairly healthy one; we, however, do not look upon the collection of our subscriptions as a duty; it is a genuine pleasure. Besides, the majority of our subscribers think so well of the JOURNAL that they usually pay for it in advance, which relieves us of the effort to get "doctors to pay promptly." Not alone do they pay in advance, but they want the JOURNAL, and if it is a mail late the telephone bells are worn to shreds of jangling metal by anxious inquirers. No, we are not tired of giving away literature. The only weariness we experience is in trying to give away some of the literature which comes to us stamped in beautiful purple letters, "sample copy." As we have already stated, we are so popular with the medical profession, that we cannot give the JOURNAL away if we wanted to; people absolutely insist on paying for it. Besides, our general air of careless prosperity gives us a financial standing, which overawes the "sample copy" fiend before he gathers himself together sufficiently to make his demand.

You have done wrong to give your journal away. It has gotten your subscribers into bad habits. We hope that next June your advertising receipts and your subscription receipts will be locked in a deadly embrace for supremacy, and that both will come out on top.

Here are some letters which one might expect to see in *The Medical Scrap,* together with appropriate answers:

### HE LIKES US.

*To the Editor of the Medical Scrap:*

We are so taken with the *Scrap* down here that we can hardly wait from one month to another for it; it is so full of good things and helps to the doctor. When I take up the *Scrap,* I feel like I used to feel when I was a boy and was reading the *Fireside Companion* back of the woodpile; I wondered if I could wait until next week for to find out whether the villain really did marry the girl, for they always stopped and "continued in our next." Do you think I should have spelled stopped, stopt? They do in some medical journals.

Dr. X.,
Lost Dog, Texas.

*Answer.*—Good. We want you to wait for the *Scrap.* It's the goods, and all the rest of the journals are dime novels compared with us. You can spell stopped any old way you wish; we let our subscribers do anything they like. You do not ask for advice about a case? Maybe you overlooked it; all our subscribers ask about cases, and you should not be backward. However, you probably have a case which puzzles you. We have used Xzlmpxdyfmhg with a great deal of success and it will probably cure your patient and he will bless you and trumpet your fame broadcast throughout the land. You will find the remedy spoken of very highly on advertising page xc.—Ed.

### A CASE FOR DIAGNOSIS.

*To the Editor of the Scrap:*

I have a patient who is in bad shape, and as I cannot find out what the matter is, I thought I would write you, as you have been so good and given so much good advice to other doctors who have written to you about cases which bothered them, and told them what to do which did it, and so I come to *The Scrap* for help in a case which bothers me a whole lot. This man is suffering with what I thought was grip with general malaise and a sinking sensation in his stomach. I gave him some atropine granules, with a few drops of belladonna tincture, every three or four hours. First, he seemed to be cured, because he did not complain of his original symptoms any more, but said his throat felt hot and dry, and I made a diagnosis of angina pectoris beginning scarlet fever; his throat was quite scarlet it seemed to me when I looked at it with one blade of my speculum which you sent me, and it has proved a very useful instrument as you said, for it is a tongue depressor and a speculum and a spoon for stirring medicines, and if you will put in just enough medicine liquida to about half an inch from the end you will have about a teaspoonful of medicine for your patient. I don't think he has scarlet fever now, because he has got quite pale and sweats some, and I come to you for help. What do you think it is? There was a disease here some years ago which baffled the best medical talent in the country, and I thought maybe this was a breaking out of another epidemic.

I like the *Scrap* better every month, and all the rest of the people like it. There was a mistake made in an address in a little town just over the county-border. One of your journals came there addressed to James Taylor, M.D. It was a sample copy. James Taylor, M.D., died about eight years ago, but there was another James Taylor there, who keeps a stable down on the reservation road, and the postmaster says he got tired of sending them back, so he gave this one to Jim Taylor, and Jim had a sickness of some sort and he looked in the *Scrap* and saw something which fitted his symptoms, so he wrote to Milwaukee for some of the stuff, and when it came, he took it and it cured him, for he died the same night. He was kind of foolish anyway and somebody told him he had bugs, and he bought some of the bug powder that you told Dr. B. would kill bugs, and Jim took it all. They think it is an awful good joke down here, and they think the *Scrap* is quite humorous.

ARTHUR M., M.D.,
Wayup, Wis.

*Answer.*—Thanks, doctor, for your kind words. Your patient is evidently a very sick man, and you will have to work hard to save him. He has evidently got a bad attack of anginitis, due to taking something which has disagreed with him. Better give him a brisk cathartic and follow it with a gentle laxative. You probably saved his life by using what you did, and he owes you a great debt, which he can never repay with coarse money. For a purgative, we would suggest the purgative pellets, mentioned incidentally on advertising page cxlviii.; for a laxative the foaming salts which we have put on the market. Let us hear from you again, doctor, and tell us how your patient came out. If he does not get any better write us again and we will suggest something else. Ha, ha! that was a joke on your friend Taylor, doctor. Can you suggest someone who would be interested in receiving the *Scrap* in your neighborhood?—Ed.

---

It is claimed by several investigators that large quantities of chloride of sodium, or better still, Trunecek's organic serum, which is composed of the different salts of the blood, given in large quantities will prevent or delay arteriosclerosis, or old age of the arteries.—*Texas Medical Gazette.*

